# Assessing the Delivery of Molecules to the Mitochondrial Matrix Using Click Chemistry

**DOI:** 10.1002/cbic.201600188

**Published:** 2016-05-25

**Authors:** Kurt Hoogewijs, Andrew M. James, Robin A. J. Smith, Michael J. Gait, Michael P. Murphy, Robert N. Lightowlers

**Affiliations:** ^1^ Medical Research Council Laboratory of Molecular BiologyCambridgeCB2 0QHUK; ^2^ Medical Research Council Mitochondrial Biology UnitCambridgeUK; ^3^ Department of ChemistryUniversity of OtagoDunedinNew Zealand; ^4^ The Wellcome Trust Centre for Mitochondrial ResearchInstitute for Cell and Molecular BiosciencesThe Medical SchoolNewcastle UniversityNewcastle upon TyneUK

**Keywords:** click chemistry, drug delivery, mitochondria, mitochondrial DNA, targeting

## Abstract

Mitochondria are central to health and disease, hence there is considerable interest in developing mitochondria‐targeted therapies that require the delivery of peptides or nucleic acid oligomers. However, progress has been impeded by the lack of a measure of mitochondrial import of these molecules. Here, we address this need by quantitatively detecting molecules within the mitochondrial matrix. We used a mitochondria‐ targeted cyclooctyne (MitoOct) that accumulates several‐ hundredfold in the matrix, driven by the membrane potential. There, MitoOct reacts through click chemistry with an azide on the target molecule to form a diagnostic product that can be quantified by mass spectrometry. Because the membrane potential‐dependent MitoOct concentration in the matrix is essential for conjugation, we can now determine definitively whether a putative mitochondrion‐targeted molecule reaches the matrix. This “ClickIn” approach will facilitate development of mitochondria‐targeted therapies.

Mitochondria are at the heart of metabolism,^1^ consequently mitochondrial dysfunction underlies multiple pathologies, and there is considerable interest in targeting probes and therapies to mitochondria.^2^ Many molecules we wish to send to mitochondria are large or polar, such as peptides, nucleic acid oligomers and polymers, and so cannot pass through membranes.^2^ These molecules require specialised delivery strategies, for example linking to a mitochondria‐targeting sequence (MTS) peptide and thereby enabling uptake through the mitochondrial protein import machinery.^2^ However, the development of mitochondrion‐targeted molecules has been hampered by the limitations of the methods used to confirm their uptake, such as confocal microscopy or cell fractionation.^3^ These methods are not sensitive enough to demonstrate that the molecules are free in the mitochondrial matrix, rather than adsorbed to the organelle's outer surface, trapped in the inter‐membrane space or stuck to the protein import machinery. As delivery to the matrix is essential for efficacy, we have developed a strategy, ClickIn, to test definitively and quantitatively whether or not a molecule is in the matrix.

Many mitochondria‐targeted molecules incorporate an MTS, therefore we first used an MTS conjugate to test the ClickIn approach. We utilised MitoOct, a mitochondria‐targeted click‐reagent,^4^ comprising a cyclooctyne linked to the lipophilic triphenylphosphonium (TPP) cation, well established to drive several‐hundredfold accumulation into the mitochondrial matrix in response to the membrane potential (Δ*ψ*_m_).^2^ The strained cyclooctyne readily undergoes cycloaddition with an azide to form a 1,2,3‐triazole by click chemistry.^5^ Therefore, a molecule containing an azido group that accesses the matrix should react there with MitoOct to form a diagnostic product that can be detected by mass spectrometry (Figure 1 A). If the molecule is not taken up by mitochondria, its reaction with MitoOct is negligible. Furthermore, conjugation can be stopped by blocking MitoOct accumulation with an uncoupler to dissipate the Δ*ψ*_m_, or by preventing the uptake of the targeted molecule (Figure 1 A).

**Figure 1:**
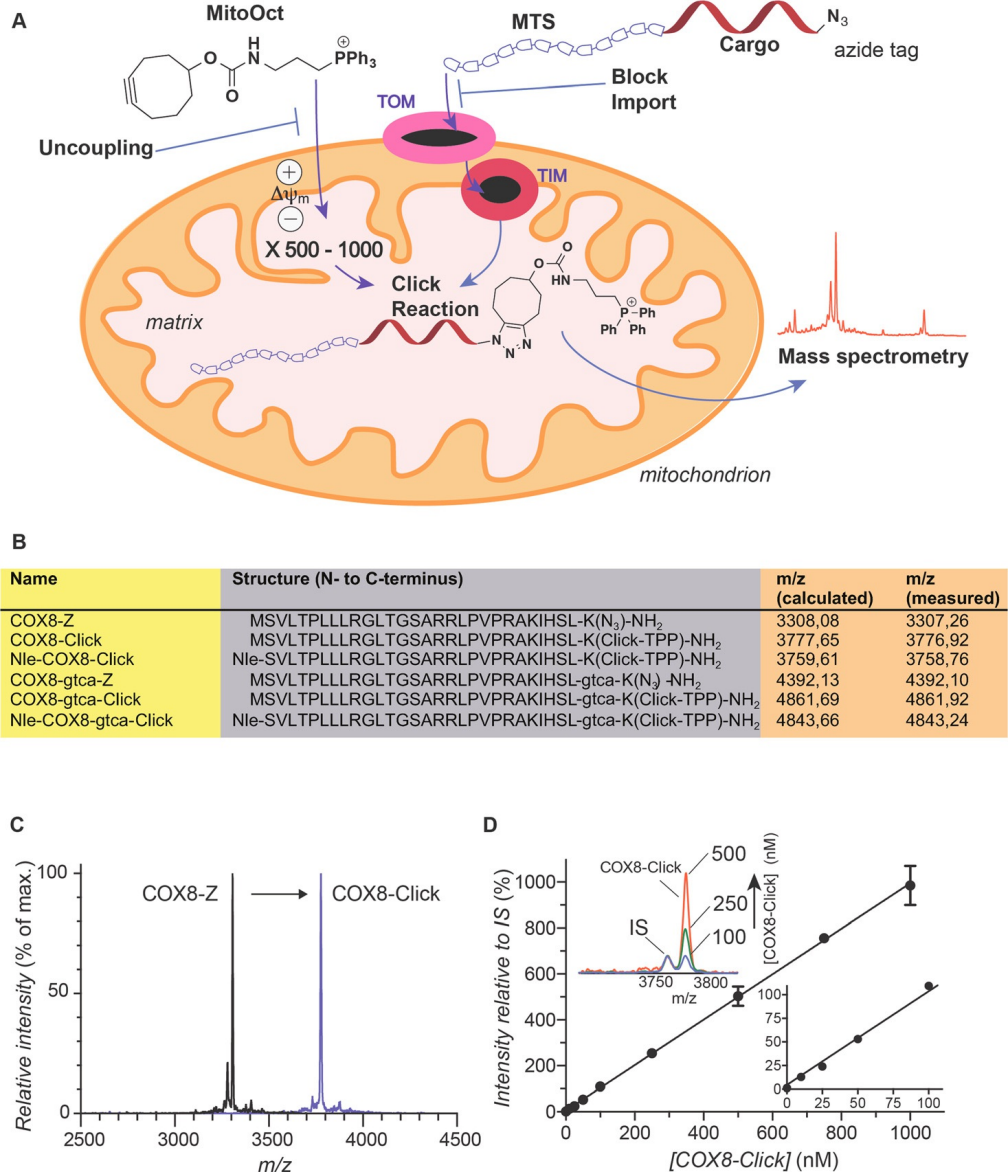
The ClickIn strategy. A) The mitochondrial membrane potential (Δ*ψ*_m_) drives MitoOct accumulation 500–1000‐fold within the matrix. The MTS‐targeted molecule, which contains an azido tag enters the mitochondrial matrix through the TOM/TIM protein import machinery and there reacts with MitoOct by a click reaction to form a product that can be measured by mass spectrometry. Adduct formation can be prevented by blocking MitoOct uptake with an uncoupler to dissipate Δ*ψ*_m_ or by using an inhibitor of the protein import machinery to prevent MTS uptake. B) Sequences and masses of the molecules used. C) MALDI‐ToF spectra for COX8‐Z and its product COX8‐Click. D) Standard curve showing the intensity observed by MALDI‐ToF for COX8‐Click normalised to that of the internal standard (IS). The upper inset shows some of the MALDI‐ToF spectra used to generate the standard curve. The lower inset is an expansion of the standard curve at a lower concentration of COX8‐Click.

To assess this approach we used the 29‐residue MTS of the COX8 cytochrome *c* oxidase subunit, widely used to target molecules to mitochondria through the protein import machinery.^6^ This peptide (COX8‐Z) was synthesised by standard Fmoc solid‐phase peptide synthesis with azidolysine (Z) at the C terminus to introduce a clickable azido group (Figure 1 B). Reaction of COX8‐Z with MitoOct formed COX8‐Click, which could be detected by MALDI‐ToF MS (Figure 1 C). We made an internal standard (IS) by replacing the N‐terminal Met with norleucine (Nle) to generate Nle‐COX8‐Z, which made Nle‐COX8‐Click upon reaction with MitoOct (Figure 1 B). A standard curve was prepared from a range of COX8‐Z‐Click concentrations, and the MS response to COX8‐Click was normalised relative to that of the IS (Figure 1 D).

To assess uptake, both COX8‐Z and MitoOct were incubated with mitochondria, and a Δ*ψ*_m_ was generated by succinate oxidation (Figure 2 A). The click reaction was terminated with excess 3‐phenyl‐1,2,4,5‐tetrazine (PhTet),^4^ which reacts ∼10^8^ times faster with cyclooctyne than does the azido tag on COX8‐Z.^4^, ^7^ The mitochondria were then pelleted, extracted and analysed by MALDI‐ToF MS (Figure 2 A). This showed COX8‐Click formation through reaction of COX8‐Z with MitoOct (Figure 2 B, inset). After 15 min ∼16 % of added COX8‐Z was converted to COX8‐Click. Importantly, dissipating Δ*ψ*_m_ with the uncoupler carbonyl cyanide‐4‐(trifluoromethoxy)phenylhydrazone (FCCP), completely blocked COX8‐Click formation, consistent with the formation of COX8‐Click in the matrix. COX8‐Z accumulation in mitochondria (along with a −28 Da form due to N_2_ neutral loss) was also blocked by FCCP (Figure 2 B). There was an increase in COX8‐Click over time that could also be prevented by FCCP (Figure 2 C).

**Figure 2:**
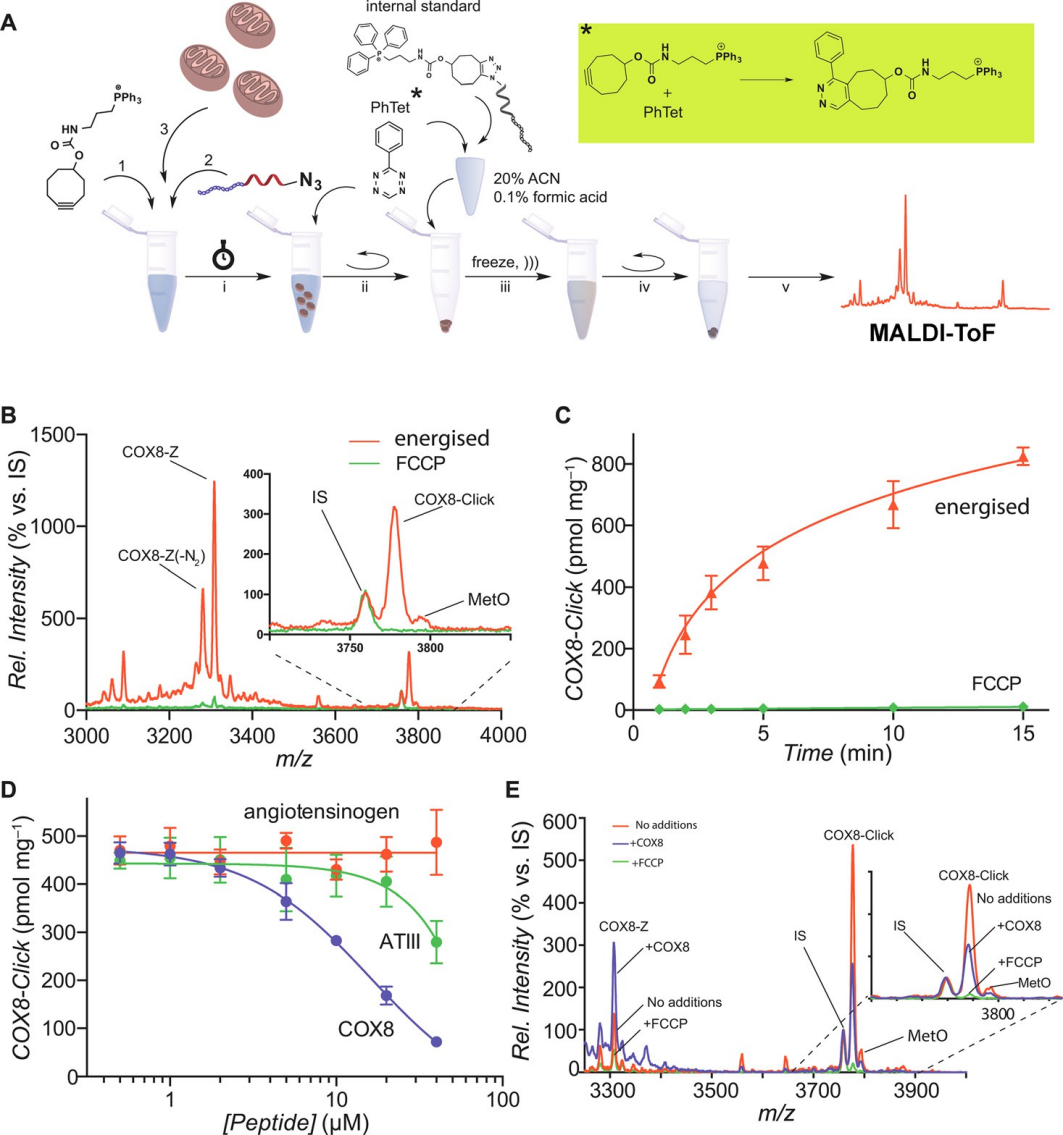
A) Application of the ClickIn strategy to mitochondria. MitoOct (1), azido‐labelled mitochondria‐targeted molecule (2) and mitochondria (3) are mixed and i) incubated, after which PhTet is added (* reacts with remaining MitoOct). ii) Next mitochondria are pelleted, and the supernatant is removed. iii) The pellet is extracted, with addition of the internal standard and more PhTet followed by freeze–thaw/sonication. iv) Then debris is precipitated, and v) the supernatant is analysed by MALDI‐ToF. B) MALDI‐ToF spectra of COX8‐Click formation in mitochondria. Mitochondria (1 mg protein/mL) were incubated in import buffer with COX8‐Z (5 μm) and MitoOct (10 μm) for 2 min, extracted and analysed. C) Time course of the formation of COX8‐Click in mitochondria. Incubations were carried out as in (B). D) Effect of peptides on COX8‐Click formation in mitochondria. Incubations were carried out as in (B) for 15 min in the presence of the indicated concentrations of COX8, angiotensinogen (1–14) (DRVYIHPFHLLVYS) or ATIII peptide (RNASVLKSSKNAKRYLRCNLKA). E) MALDI‐ToF spectra of COX8‐Click formation in mitochondria, as described in (D), ±COX8 peptide (10 μm) or FCCP. Data in (C) and (D) are mean±SEM for three independent experiments. The peaks marked MetO in (B) and (E) are due to methionine oxidation

To confirm that COX8‐Z enters mitochondria by using the protein import machinery, we added excess COX8 lacking the C‐terminal azidolysine, to compete with COX8‐Z uptake. Increasing the concentration of COX8 decreased COX8‐Click formation (Figure 2 D). In contrast, the control peptides angiotensinogen (1–14) and ATIII peptide, which are not taken up by mitochondria,^8^ did not affect COX8‐Click formation at concentrations up to 20 μm; this is consistent with COX8‐Z uptake through the protein import machinery (Figure 2 D). As Δ*ψ*_m_ is unaffected by these peptides, the lack of COX8‐Click formation is due to preventing the uptake of COX8‐Z, not MitoOct. We conclude that the ClickIn approach can confirm that a putative mitochondria‐targeted molecule is within the matrix.

Among the many bioactive molecules we seek to target to mitochondria are those that interact with mitochondrial DNA (mtDNA); as this is essential for mitochondrial function,^9^ mutations to mtDNA cause a number of diseases.^10^ Considerable efforts are ongoing to develop molecules that alter mtDNA function in a sequence specific way, either as probes or as potential therapies.^2^ These agents include DNA‐mimetic oligomers, such as peptide nucleic acids (PNAs), that bind selectively to complementary DNA sequences.^11^ Therefore as a proof of principle, we assessed the mitochondrial uptake of a COX8 peptide conjugated to a PNA tetramer (GTCA) followed by a C‐terminal azide (COX8‐PNA‐Z) (Figure 1 B). COX8‐PNA‐Z forms COX8‐PNA‐Click after treatment with MitoOct (Figure 3 A). The corresponding IS was generated by replacing the N‐terminal Met with Nle (Nle‐COX8‐PNA‐Click, Figure 1 B). The mitochondrial uptake of COX8‐PNA‐Z was assessed in the same way as for COX8‐Z. The formation of COX8‐PNA‐Click was shown by MALDI‐ToF MS; although this was blocked by FCCP (Figure 3 B). A time course showed an increase in COX8‐PNA‐Click (Figure 3 C). When the FCCP‐sensitive formation of COX8‐PNA‐Click was assessed following incubation with a range of COX‐PNA‐Z concentrations, COX8‐PNA‐Click formation showed saturation behaviour consistent with the uptake of COX‐PNA‐Z through the mitochondrial protein import machinery. Therefore, the ClickIn approach can assess the uptake of bioactive mitochondria‐targeted molecules.

**Figure 3:**
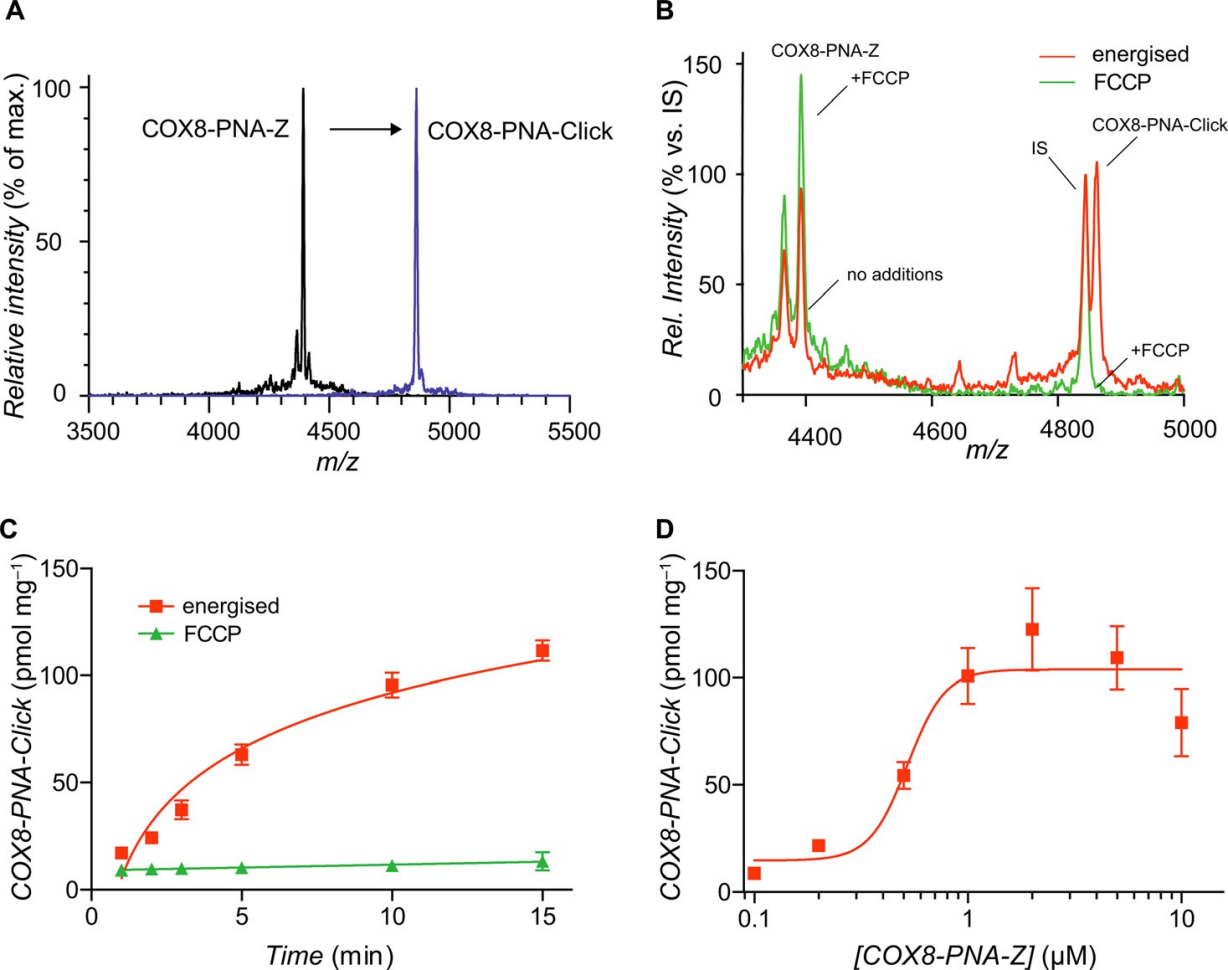
ClickIn assessment of the mitochondrial uptake of PNA tetramer by conjugation to COX8. A) MALDI‐ToF MS of COX8‐PNA‐Z and its click product COX8‐PNA‐Click formed by reaction with MitoOct. B) MALDI‐ToF spectra of COX8‐PNA‐Click formation upon incubation of 2 μm COX8‐PNA‐Z with mitochondria (1 mg protein/mL) and 10 μm MitoOct for 15 min ±FCCP. C) Time course of the formation of COX8‐PNA‐Click in mitochondria. Incubations were carried out as in (B). Data are means ±SEM for three experiments. D) Formation of COX8‐PNA‐Click in mitochondria incubated with different concentrations of COX8‐PNA‐Z. Incubations were carried out as in (B) for 15 min ±FCCP. Background values for +FCCP are subtracted from the total uptake. Data are mean±SEM (*n*=4).

There was a large amount of COX8‐Z associated with energised mitochondria, which was lost in the presence of FCCP, but which did not form COX8‐Click (Figure 2 B). This might be COX8‐Z that has accumulated within the mitochondrial matrix but which has yet to be converted to COX8‐Click owing to the relatively slow reaction of MitoOct with an azide (∼0.1–0.2 m ^−1^ s^−1^).^4^ However, in the presence of COX8, the amount of COX8‐Z associated with the mitochondrial pellet increased, whereas COX8‐Click formation decreased (Figure 2 E). Similarly, the amount of COX8‐PNA‐Z associated with the mitochondrial pellet increased in the presence of FCCP, which completely blocked formation of COX8‐PNA‐Click (Figure 3 B). These examples illustrate that the association of a putative targeted molecule with mitochondria is an unreliable and misleading indication of its matrix import and demonstrate the utility of the ClickIn approach.

The uptake of bioactive molecules to mitochondria is a promising approach to reporting on function and to developing therapies.^2^ However, the targeting of large polar molecules to mitochondria has not yet fulfilled its potential, in part because the methods used to assess their uptake have not shown definitively that the construct is in the mitochondrial matrix. The ClickIn method enables progress by confirming that the molecule has been taken up by mitochondria. The ClickIn strategy will allow us to test a range of strategies for the uptake of diverse molecules into isolated mitochondria. Future work will extend this approach to assess uptake of molecules into mitochondria within cells and in vivo, as the MitoOct Click reaction occurs readily within mitochondria in cells and in vivo.^4^ A further use of the ClickIn approach is to assess uptake of molecules such as RNA into mitochondria, a contentious and exciting research area,^12^ by attaching an azido group to the molecule. In summary, we have introduced the ClickIn strategy, which will enable us to progress rationally with assessing and optimising the delivery of large and polar reagents to mitochondria and thereby provide a heuristic tool to develop therapies for mitochondrial disorders.

## Supporting information

As a service to our authors and readers, this journal provides supporting information supplied by the authors. Such materials are peer reviewed and may be re‐organized for online delivery, but are not copy‐edited or typeset. Technical support issues arising from supporting information (other than missing files) should be addressed to the authors.

Supplementary
