# Supplementary material for: Assessing the Delivery of Molecules to the Mitochondrial Matrix Using Click Chemistry
Source: Chembiochem. 2016 May 25;17(14):1312–6. doi: 10.1002/cbic.201600188 (PMC4973694; doi:10.1002/cbic.201600188)
Supplement: Supplementary file 1 — Supplementary [file CBIC-17-1312-s001.pdf]

CHEMBIOCHEM

Supporting Information

Assessing the Delivery of Molecules to the Mitochondrial Matrix Using Click Chemistry

Kurt Hoogewijs,[a, b, d] Andrew M. James,[b] Robin A. J. Smith,[c] Michael J. Gait,*[a] Michael P. Murphy,*[b] and Robert N. Lightowlers*[d]

cbic_201600188_sm_miscellaneous_information.pdf

SUPPORTING INFORMATION

MATERIALS AND METHODS

Materials

Synthesis of 3-(cyclooctyn-4-yloxycarbonylamino)propyl triphenylphosphonium bromide (MitoOct) and 3-phenyl-1,2,4,5-tetrazine (PhTet) was previously described [1]. Fmoc protected amino acids were obtained from AGTC Bioproducts or Iris Biotech, Fmoc protected PNA-monomers were obtained from LinkTech, coupling reagents N,N'-diisopropylcarbodiimide (DIC), ethyl (hydroxyimino)cyanoacetate (Oxyma), (benzotriazol-1-yloxy)tripyrrolidinophosphonium hexafluorophosphate (PyBOP) and N,N-diisopropylethylamine (DIPEA) were obtained from AGTC Bioproducts or Sigma-Aldrich, solvents were obtained from Alfa Aesar, Sigma-Aldrich and AGTC. All other reagents were obtained from Sigma-Aldrich. RP-HPLC was performed on a Varian 940-LC with 0.1% TFA in H_2O (buffer A) and 0.1% TFA in acetonitrile (buffer B) using a Phenomenex Luna 10 µm C18 100Å 250 x 10 mm LC column for purification (4 mL per min; 3 min 0% B, 0-75% B in 20 min, 75-100% B in 1 min, 100% B for 2 min, 100-0% B in 2 min) and a Luna 5 µm C18 100Å 250 x 4.6 mm LC column for analysis (1 mL per min; 3 min 0% B, 0-100% B in 15 min, 100% B for 4 min, 100-0% B in 1 min). Sonication was done in a Grant XB2 ultrasonic bath.

Methods

Synthesis of peptides and peptide nucleic acids Peptides and PNA were synthesized on a CEM Liberty Blue peptide synthesizer on H-Rink amide ChemMatrix® resin. Fmoc amino acids were coupled using DIC/Oxyma as coupling reagents at 75°C (or 50°C for Fmoc-His(Trt)-OH) using microwave irradiation and unreacted amine groups were capped using a 5% Ac_2O and 6% lutidine solution in DMF. The Fmoc-protecting group was removed with 20% piperidine at room temperature for PNA and 75°C for peptides. Alternatively, an Intavis peptide synthesizer was used, with PyBOP/DIPEA as coupling reagents. The peptides were deprotected using TFA /3,6-dioxa-1,8-octanedithiol (DODT) /H_2O/ triisopropylsilane (TIS) (94:2,5:2,5:1) for 3 h and precipitated with diethylether. The crude peptide was purified by RP-HPLC and pure fractions were identified by MALDI-TOF and collected. For the preparation of the internal standards, 50 nmol peptide in MQ water was combined with 60 nmol

MitoOct ethanol. The mixture was evaporated *in vacuo*, and redissolved in 200 µL methanol. This procedure was repeated, typically 5 times, until the reaction was complete as assessed by MALDI-TOF mass spectrometry and HPLC. Purification by RP-HPLC yielded the internal standards (IS) in high purity. Peptides and peptide-PNA conjugate sequences and masses are presented in Fig. 1B.

Mass spectrometry Mass spectrometry was performed on an Applied Biosystems Voyager-DE PRO MALDI-ToF spectrometer. The sample was spotted on the MALDI-plate using the bottom-layer method. 0.75 µL Matrix (50% acetonitrile, 5 mg/ml α-cyano-4-hydroxycinnamic acid, 10 mM dibasic ammonium citrate, 0.1% trifluoroacetic acid (TFA)) was spotted on the plate and 0.75 µL sample was mixed in. The spot was left to dry at room temperature, after which another layer of 0.75 µL of matrix was added. In total 10 spectra with 20 shots each were collected per spot, using a minimum absolute intensity of 1000 as selection criterion, averages were taken of 3 spots. For the standard curves, 0.1 µM internal standard was mixed with different concentrations of IS in 20 % ACN supplemented with 0.1% bovine serum albumin (BSA). Spectra were processed using mMass software, and relative ion intensities compared to an IS were used for quantification (Fig. 1D).

Accelerated strain-promoted alkyne-azide cycloaddition in isolated mitochondria. Rat liver or heart mitochondria were prepared by differential centrifugation as previously described [2]. Preliminary experiments showed that COX8-Z was rapidly degraded by the MTS processing peptidases within the mitochondrial matrix and also by proteases in the mitochondrial preparation. Therefore a range of protease inhibitors was used to inhibit these reactions [3].

Mitochondria (20 mg protein /ml) were incubated on ice with 2 mM phenanthroline, 5 mM EDTA in STE buffer (250 mM sucrose, 5 mM Tris, 1 mM EGTA, pH 7.4) for 15 min before addition to the reaction mixture to a final concentration of 1 mg protein/ml in 100 µl KCl buffer (120 mM KCl, 10 mM HEPES, 1 mM EGTA, 1 mM ATP, 1 mM $MgCl_2$, 0.05 % BSA, 2 mM phenanthroline and complete protease inhibitor (EDTA free), pH 7.4) supplemented with 10 mM potassium succinate, 4 µg/ml rotenone and 1µM nigericin. Unless otherwise specified, the mitochondria were incubated at 37ºC for 15 min, after which the reaction was stopped by addition of PhTet (50 µM). The mitochondria were then pelleted by

centrifugation (1 min at 16000 x g), after which the supernatant was removed. To the pellet was added 100 µL 20% acetonitrile (ACN) containing 0.1 % formic acid, 50 µM 3-phenyl-1,2,4,5-tetrazine (PhTet) and 0.1 µM of the appropriate internal standard (IS). Samples were frozen in dry ice, and subjected to 3 cycles of sonication followed by freezing on dry ice. The samples were centrifuged twice (16000 x g for 10 min). The supernatant was then removed and prepared for MALDI-ToF as described above. In total 10 spectra with 20 shots each were collected per spot, using a minimum intensity of 1000 as selection criterion, averages were taken of 3 spots per experiment. We assessed the formation of COX8-Click as shown in figure 2, as a function of MitoOct and COX8-Z concentrations and found that it was maximal at [COX8-Z] of ~5-10 µM and [MitoOct] of ~10 µM (data not shown).

SUPPORTING DATA

ClickIn in isolated rat heart mitochondria.

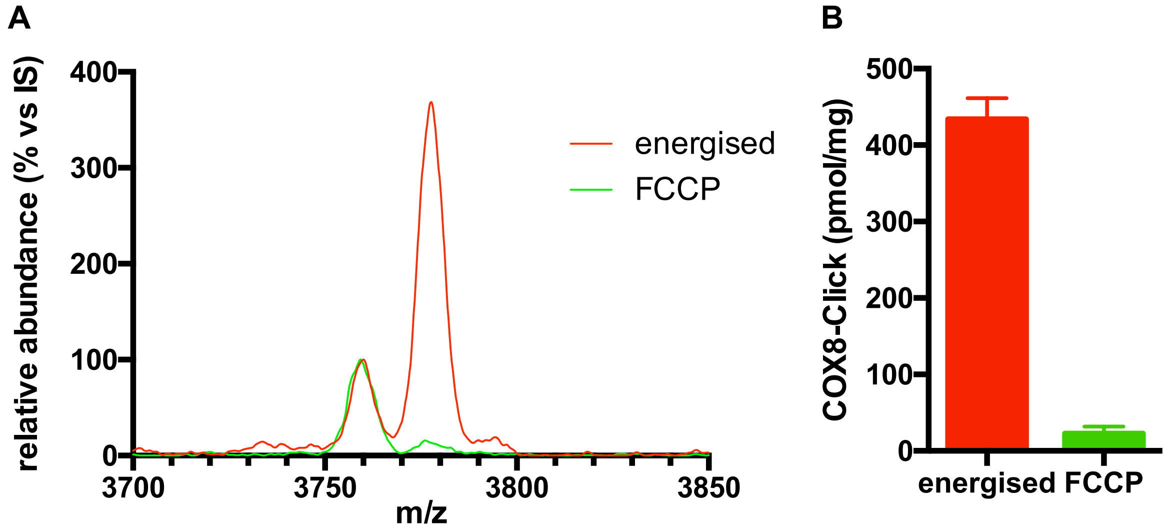

Experiments were performed as described for rat liver mitochondria in figure 2, using 5 μM COX8-Z and 10μM MitoOct for 15 min. (A) Shows typical mass spectra for energised mitochondria, and mitochondria treated with FCCP. (B) Values obtained for 2 independent experiments ± range.

CHARACTERISATION OF PEPTIDES

Nomenclature

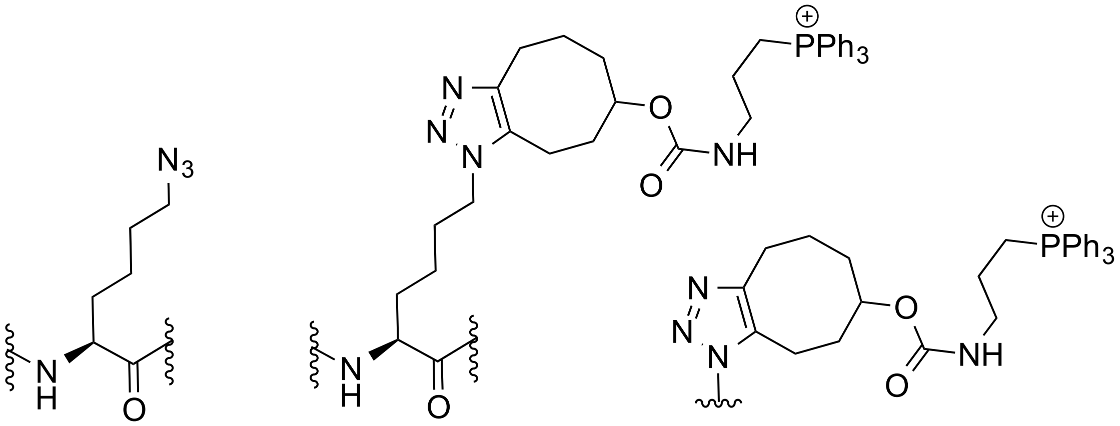

Z **Click** **Click-TPP**

COX8-Z

MSVLTPLLLRGLTGSARRLPVPRAKIHSL-K(N_3)-NH_2
Chemical Formula: $C_{147}H_{261}N_{49}O_{35}S$

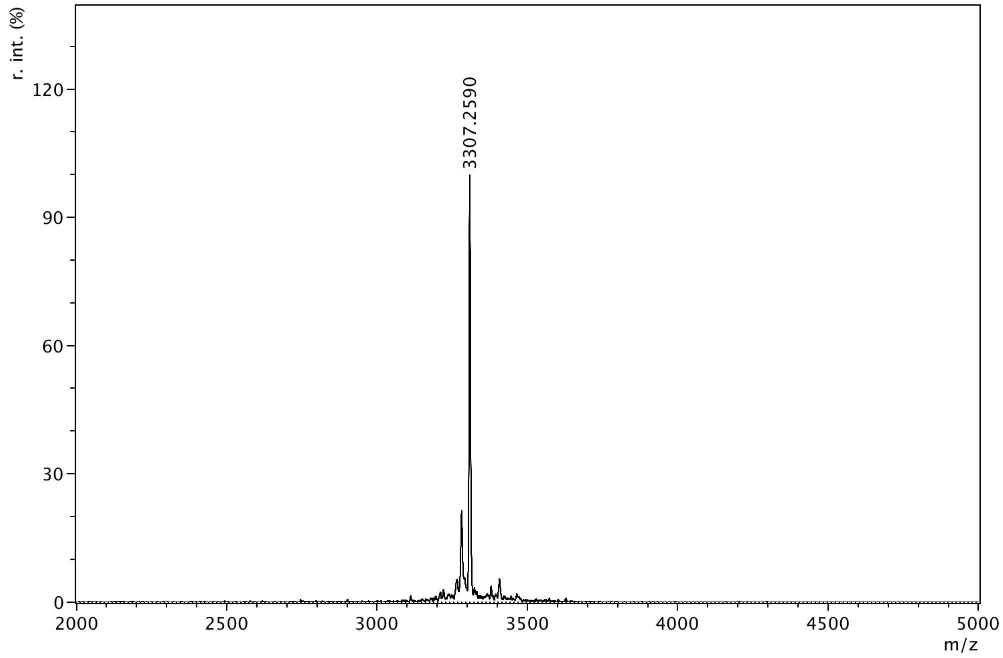

m/z (calculated) = 3308.08 (average) / 3307.00 (100%)
m/z (measured) = 3307.26

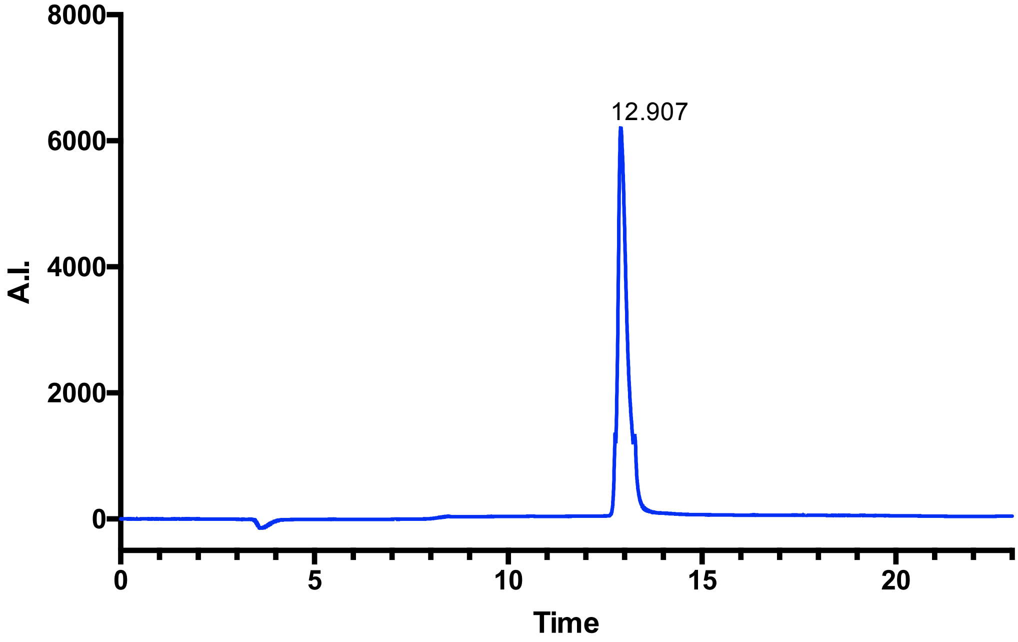

RP-HPLC Chromatogram at 205 nm, t_R = 12.90 min

COX8-Click

MSVLTPLLLRGLTGSARRLPVPRAKIHSL-K(Click-TPP)-NH_2
Chemical Formula: $C_{177}H_{294}N_{50}O_{37}PS^+$

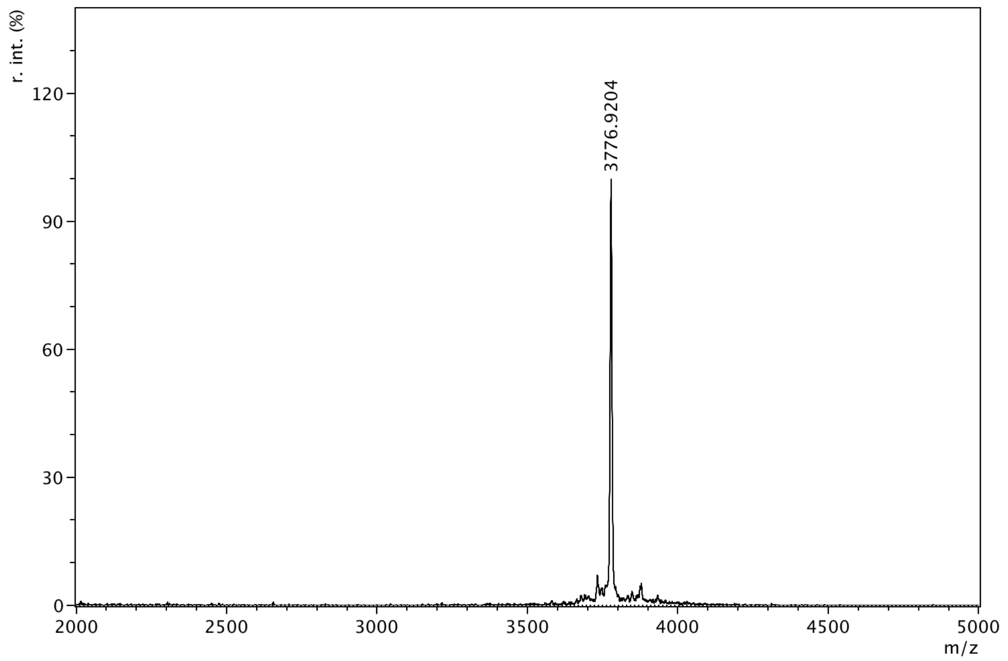

m/z (calculated) = 3777.65 (average) / 3776.22 (100.0%)
m/z (measured) = 3776.92

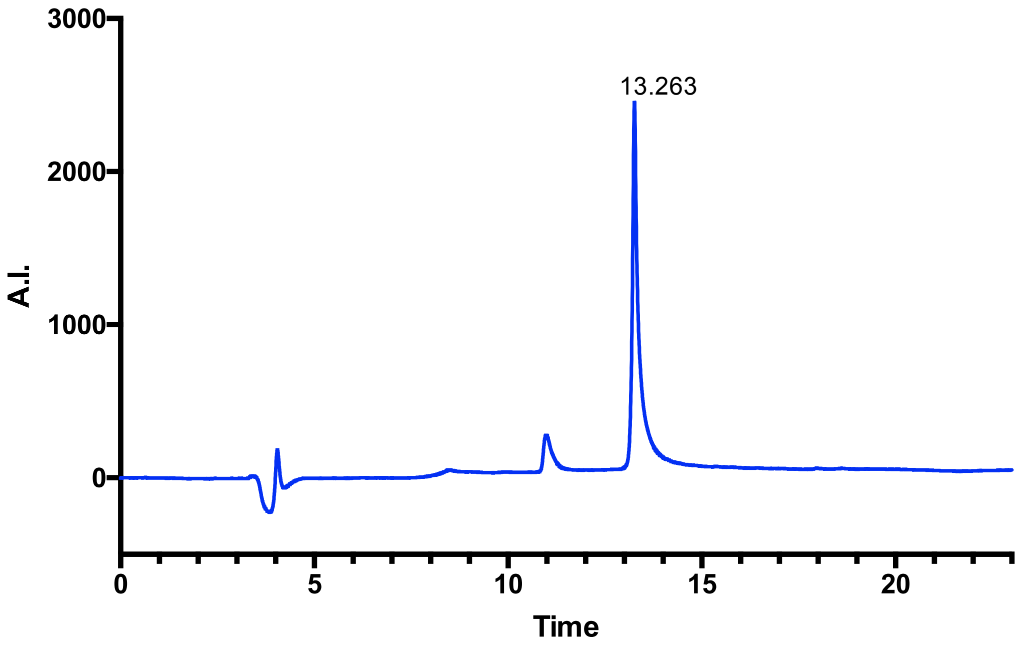

RP-HPLC Chromatogram at 205 nm, t_R = 13.26 min

Nle-COX8-Click

Nle-SVLTPLLLRGLTGSARRLPVPRAKIHSL-K(Click-TPP)-NH_2
Chemical Formula: $C_{178}H_{296}N_{50}O_{37}P^+$

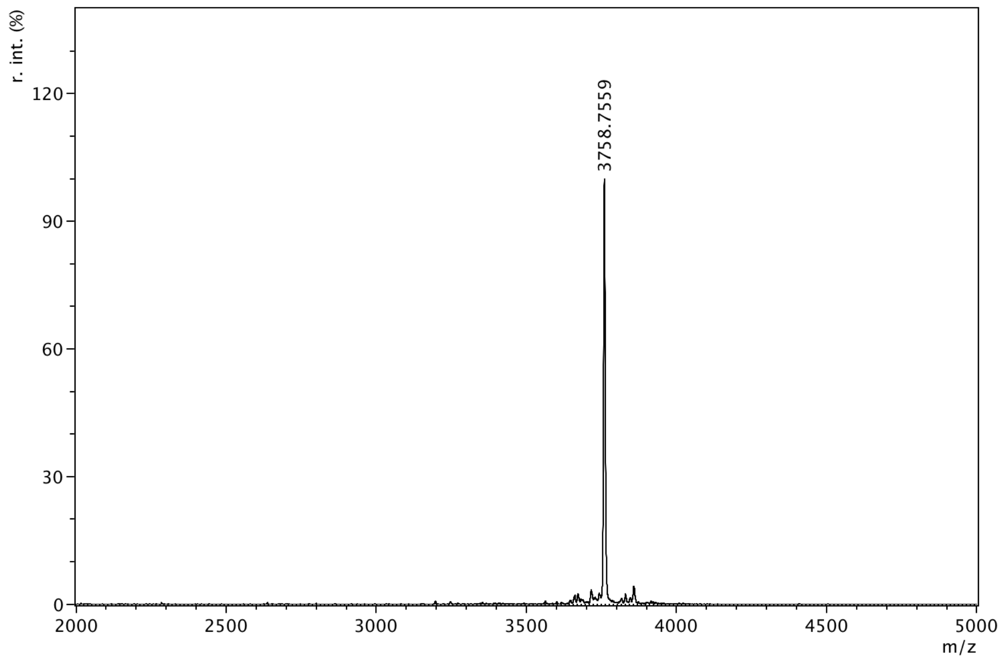

m/z (calculated) = 3759.61 (average) / 3759.26 (100.0%)
m/z (measured) = 3758.76

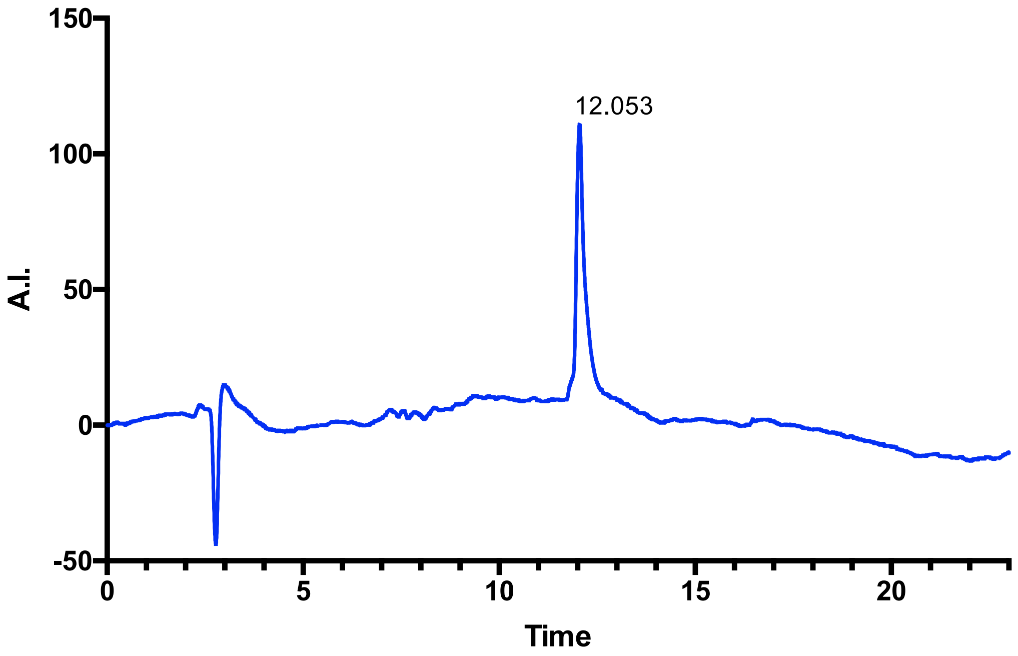

RP-HPLC Chromatogram at 205 nm, t_R = 12.05 min

COX8-PNA-Z

MSVLTPLLLRGLTGSARRLPVPRAKIHSL-GTCA-K(N_3)-NH_2
Chemical Formula: $C_{190}H_{314}N_{72}O_{47}S$

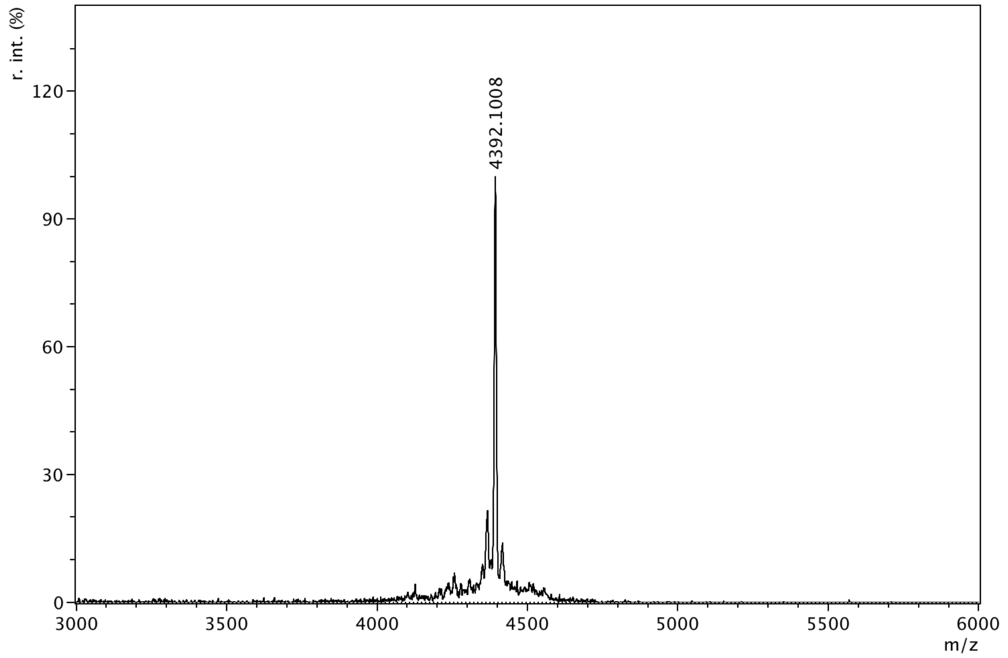

m/z (calculated) = 4392.13 (average) / 4390.42 (100.0%)
m/z (measured) = 4392.10

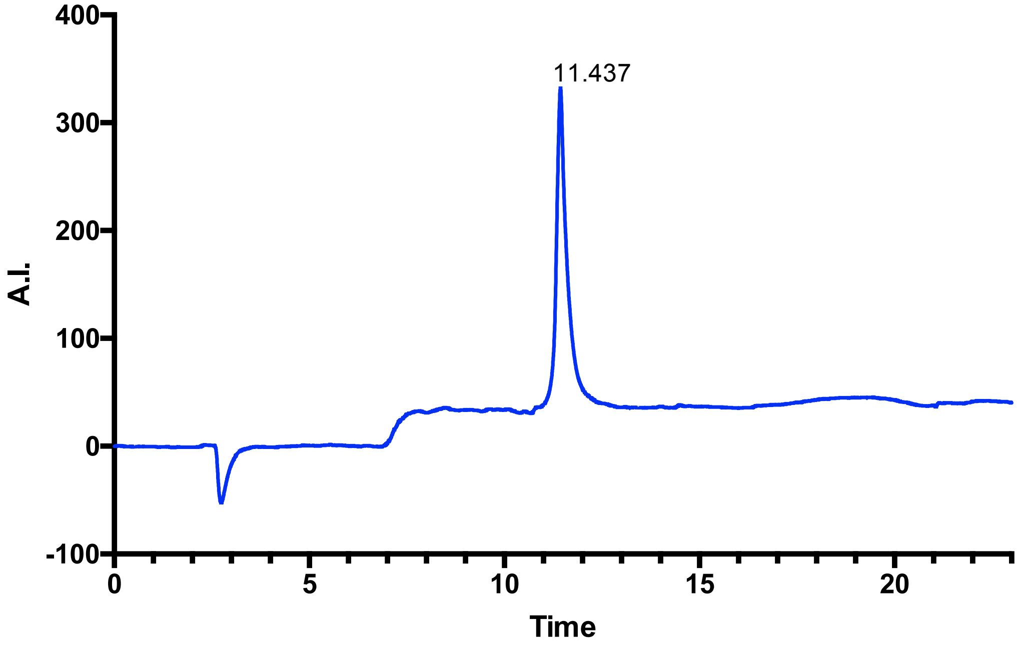

RP-HPLC Chromatogram at 205 nm, t_R = 11.44 min

COX8Z-PNA-Click

MSVLTPLLLRGLTGSARRLPVPRAKIHSL-GTCA-K(Click-TPP)-NH_2
Chemical Formula: $C_{220}H_{347}N_{73}O_{49}PS^{+}$

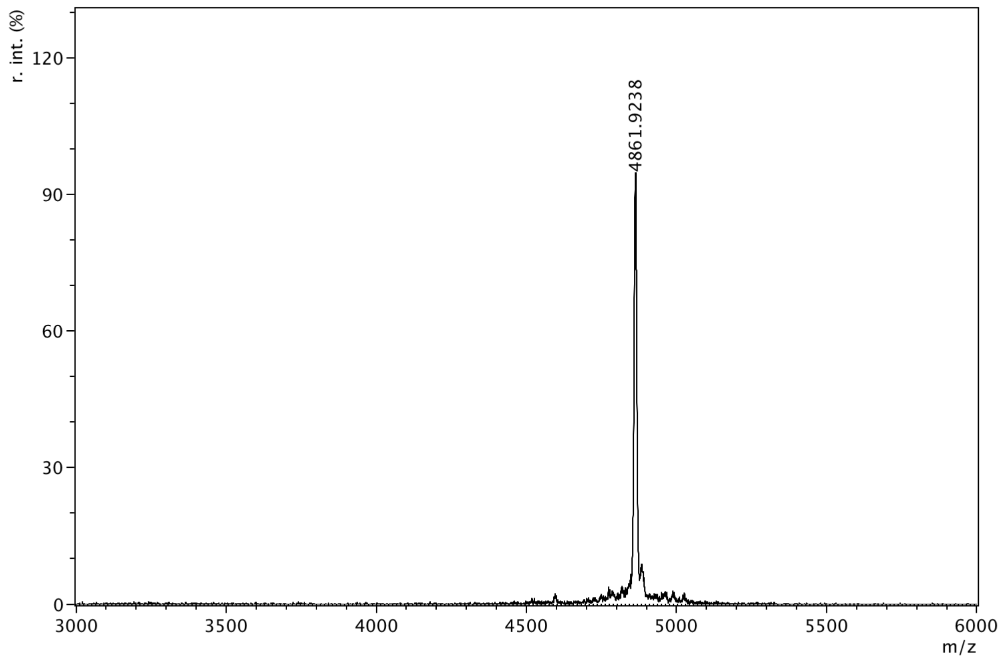

m/z (calculated) = 4861.69 (average) / 4860.64 (100.0%)
m/z (measured) = 4861.92

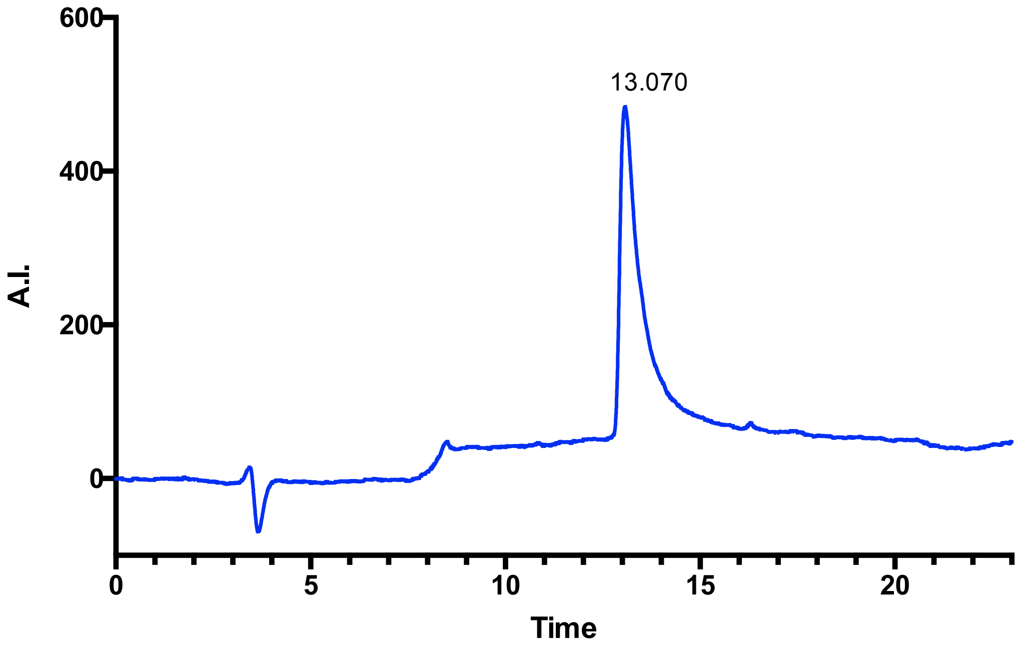

RP-HPLC Chromatogram at 205 nm, t_R = 13.07 min

Nle-COX8Z-PNA-Click

Nle-SVLTPLLLRGLTGSARRLPVPRAKIHSL-GTCA-K(Click-TPP)-NH_2
Chemical Formula: $C_{221}H_{349}N_{73}O_{49}P^+$

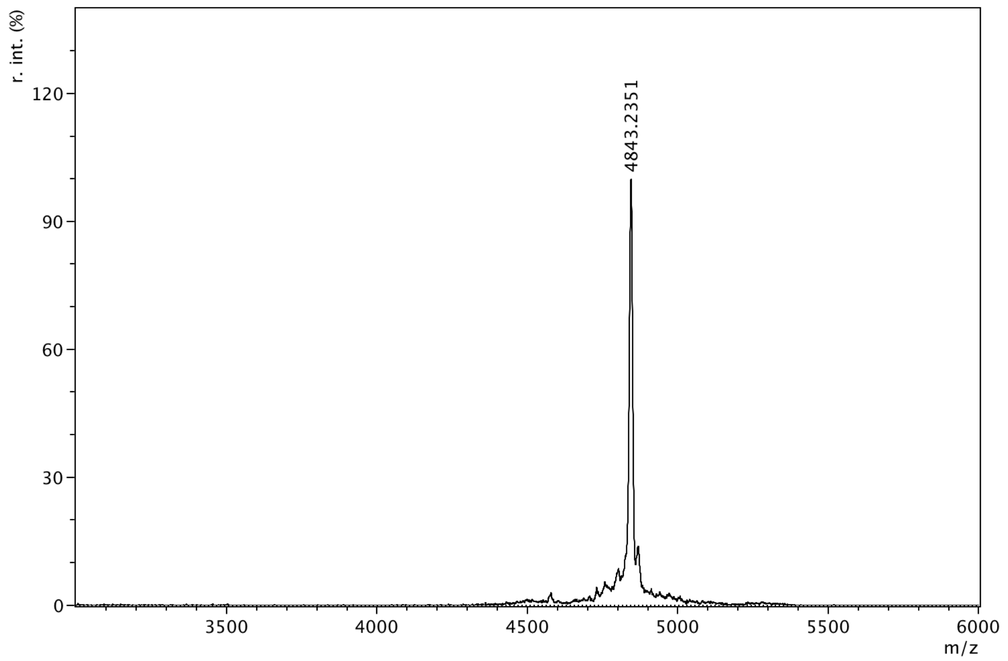

m/z (calculated) = 4843.66 (average) / 4842.69 (100.0%)
m/z (measured) = 4843.24

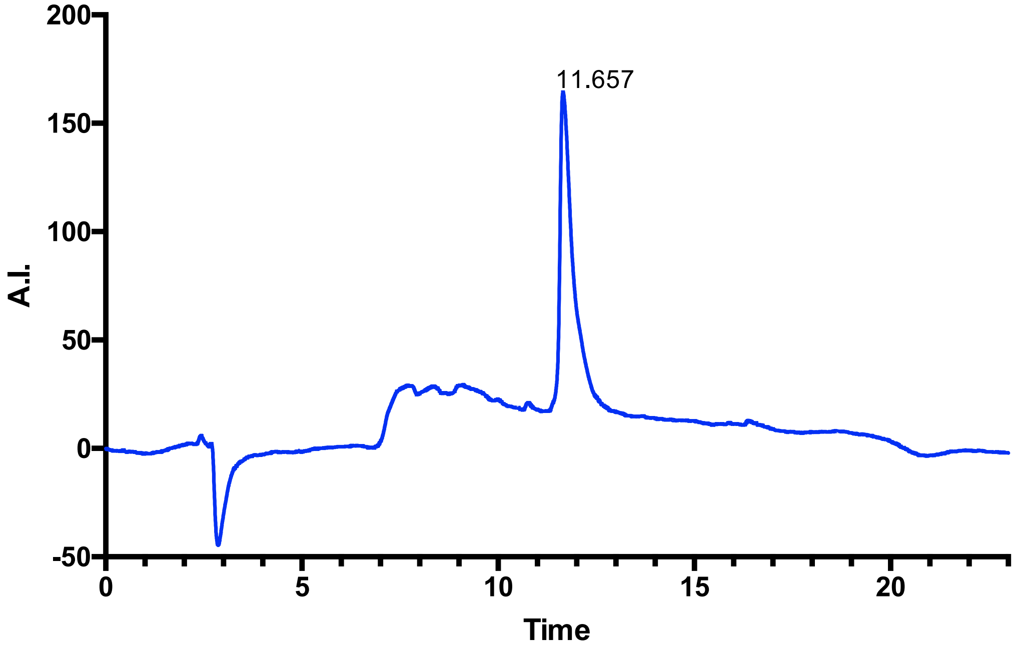

RP-HPLC Chromatogram at 205 nm, t_R = 11.66 min

COX8

MSVLTPLLLRGLTGSARRLPVPRAKIHSL-NH_2
Chemical Formula: $C_{141}H_{251}N_{45}O_{34}S$

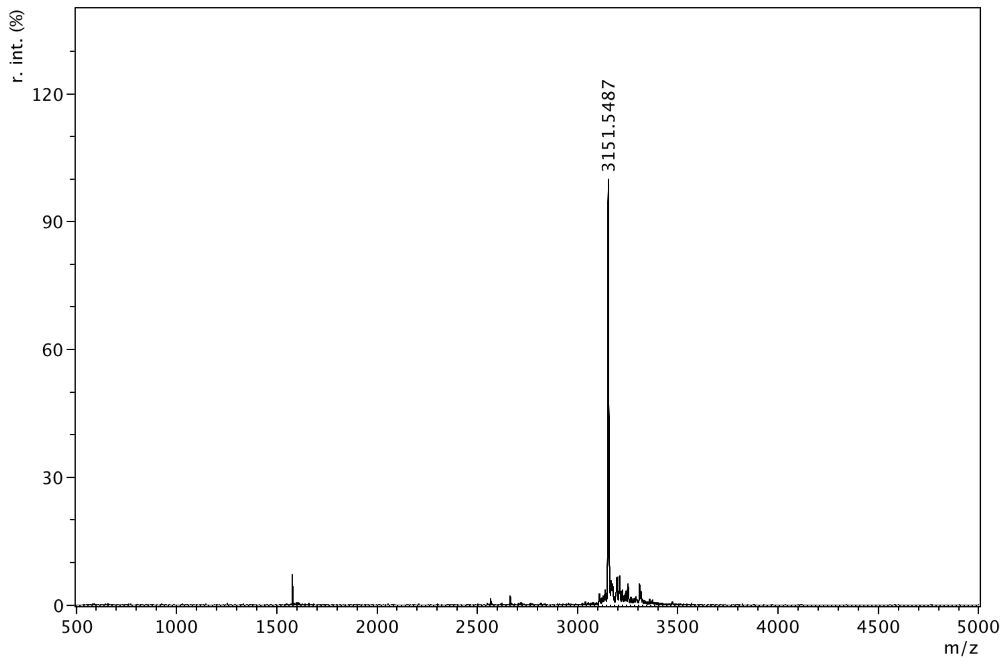

m/z (calculated) = 3153.91 (average) /3152.91 (100%)
m/z (measured) = 3151.55

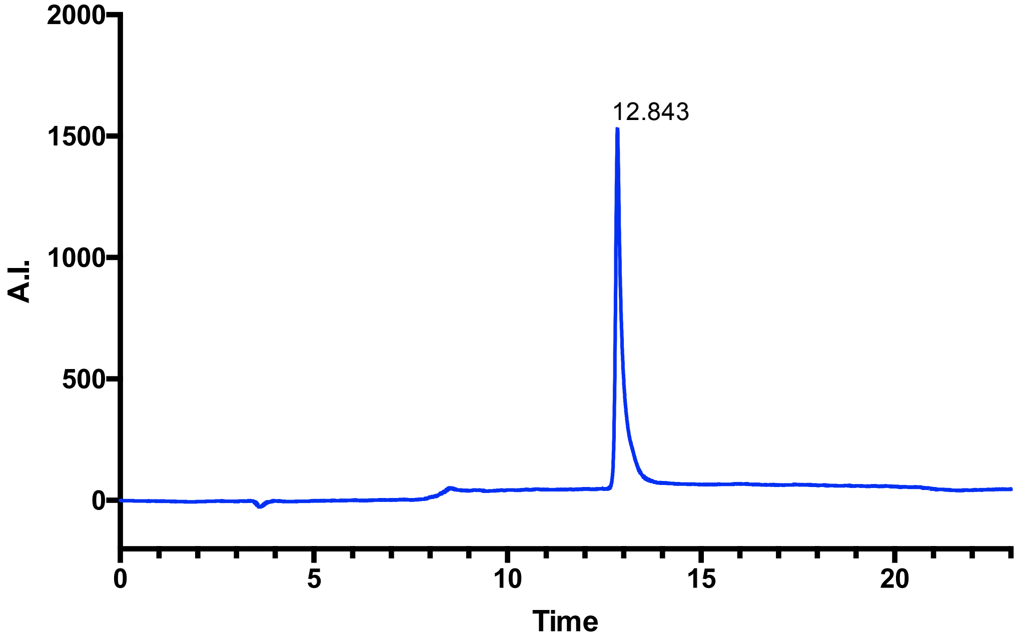

RP-HPLC Chromatogram at 205 nm, t_R = 12.84 min

ATIII

RNASVLKSSKNAKRYLRCNLKA-NH_2
Chemical Formula: $C_{107}H_{191}N_{39}O_{29}S$

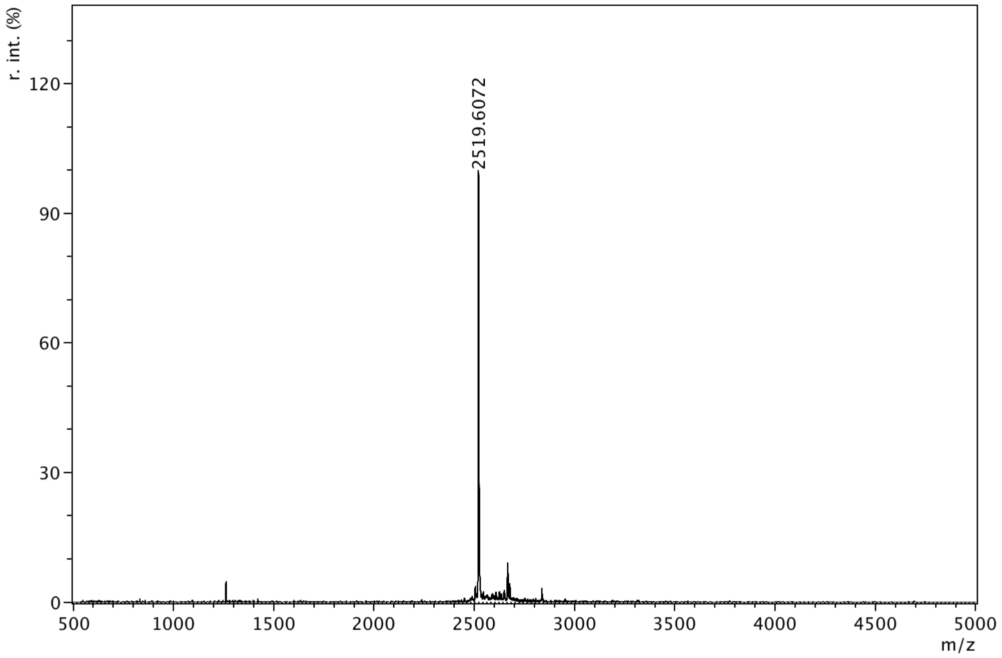

m/z (calculated) = 2521.02 (average) / 2520.45 (100%)
m/z (measured) = 2519.61

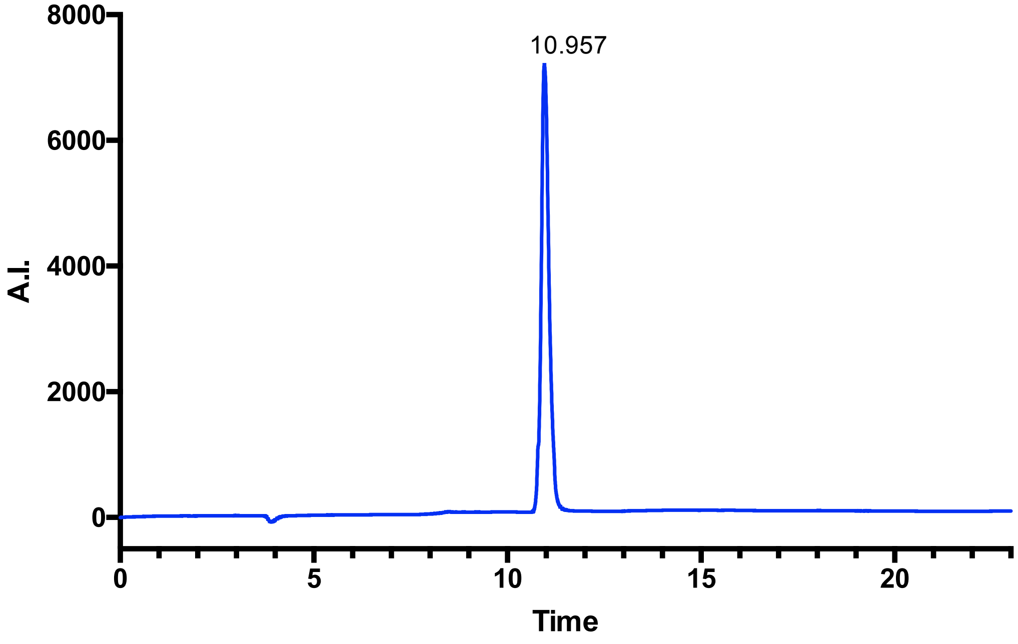

RP-HPLC Chromatogram at 205 nm, t_R =10.957 min

Angiotensinogen (1-14) DRVYIHPFHLLVYS was obtained from Sigma Aldrich (renin substrate porcine, SCP0021), and used as such.

REFERENCES

[1] A. Logan, V. R. Pell, K. J. Shaffer, C. Evans, N. J. Stanley, E. L. Robb, T. A. Prime, E. T. Chouchani, H. M. Cocheme, I. M. Fearnley, S. Vidoni, A. M. James, C. M. Porteous, L. Partridge, T. Krieg, R. A. Smith, M. P. Murphy, *Cell Metab.* **2016**, *23*, 379-385.

[2] J. B. Chappell, R. G. Hansford, in *Subcellular components: preparation and fractionation* (Ed.: G. D. Birnie), Butterworths, London, **1972**, pp. 77-91.

[3] Y. K. Pak, H. Weiner, *J. Biol. Chem.* **1990**, *265*, 14298-14307.
